# Using Pupillometry to Evaluate Balance in Patients Implanted with a Cochleo-Vestibular Implant

**DOI:** 10.3390/jcm13133797

**Published:** 2024-06-28

**Authors:** Joyce Tang, Ángel Ramos de Miguel, Juan Carlos Falcón González, Silvia Borkoski Barreiro, Isaura Rodriguez Montesdeoca, Ángel Ramos Macías

**Affiliations:** 1 Department of Otorhinolaryngology, Head and Neck Surgery, Singapore General Hospital, Singapore 169608, Singapore; joyce.tang.z.e@singhealth.com.sg; 2 Hearing and Balance Laboratory, Las Palmas de Gran Canaria University, Institute of Intelligent System and Numeric Application in Engineering, 35016 Las Palmas, Spain; ramosorl@idecnet.com; 3 Department of Otolaryngology, Head and Neck Surgery, Complejo Hospitalario Universitario Insular Materno Infantil de Gran Canaria, 35016 Las Palmas, Spain; jfalgond@gobiernodecanarias.org (J.C.F.G.); silviaborkoski@hotmail.com (S.B.B.); isaurar.mon@gmail.com (I.R.M.)

**Keywords:** cochleo-vestibular implant, pupillometry, balance, posture, bilateral vestibular hypofunction, vestibulopathy

## Abstract

Maintaining balance comes naturally to healthy people. In subjects with vestibulopathy, even when compensated, and especially if it is bilateral, maintaining balance requires cognitive effort. Pupillometry is an established method of quantifying cognitive effort. **Background/Objectives**: We hypothesized that pupillometry would be able to capture the increased effort required to maintain posture in subjects with bilateral vestibulopathy in increasingly difficult conditions. Additionally, we hypothesized that the cognitive workload during balance tasks, indexed by pupil size, would decrease with the activation of the BionicVEST cochleo-vestibular implants. **Methods:** Subjects with a cochleo-vestibular implant as of March 2023 were recruited, excluding those with ophthalmological issues that precluded pupillometry. Pupillometry was performed using a validated modified videonystagmography system. Computed dynamic posturography and a Modified Clinical Test of Sensory Integration on Balance were performed while the pupil was recorded. Tests were first performed after 24 h of deactivating the vestibular component of the implant. Thereafter, it was reactivated, and after 1 h of rest, the tests were repeated. The pupil recording was processed using custom software and the mean relative pupil diameter (MRPD) was calculated. **Results:** There was an average of 10.7% to 24.2% reduction in MRPD when the vestibular implant was active, with a greater effect seen in tasks of moderate difficulty, and lesser effect when the task was easy or of great difficulty. **Conclusions:** Despite technical challenges, pupillometry appears to be a promising method of quantifying the cognitive effort required for maintaining posture in subjects with bilateral vestibulopathy before and after vestibular implantation.

## 1. Introduction

The pupil size changes [1] reflexively in response to changes in lighting conditions (pupillary light and dark reflex), and the need for near–far accommodation (pupillary accommodation reflex). These changes are also consensual (consensual pupillary reflex) meaning that when one eye is exposed to changes in light or a need for accommodation, the contralateral pupil size changes as well [2]. These changes in the pupil size are gross, in the order of a few millimeters, and are often readily observed by the naked eye. The pupil size also changes, sometimes very subtly (sub-millimeter), in response to emotional states (psychosensory pupil response) [3,4]. The link between changes in pupil size and emotional arousal was first observed by the French physician and physiologist, Henri-Marie Husson in the mid-19th century [5]. The locus coeruleus, located in the pons, is part of the reticular activating system, and is the principal site of synthesis of norepinephrine in the brain. It is responsible for many of the physiological changes due to stress, including changes in the pupil size, as it has a pathway to the intermediolateral column and thence the superior cervical ganglion and finally, the dilator pupillae. It also sends inhibitory signals to the Edinger–Westphal nucleus, inhibiting the parasympathetic fibers to the sphincter pupillae [6,7,8]. Much like the light and accommodation reflexes, pupil size changes in response to emotions are not under conscious control [9]. This allows the pupils to be studied as a biomarker for cognitive workload or mental effort [10].

People with bilateral vestibulopathy have a multitude of symptoms [11]. Primarily, they have unsteadiness with upright positioning, worsened in darkness or when walking on uneven ground. This is due to reduced sensory vestibular input resulting in impaired vestibulospinal reflexes leading to increased body sway. An otolith cochleo-vestibular implant provides constant electrical stimulation of the inferior vestibular nerve in subjects with bilateral vestibulopathy by stimulating the region of the saccule. Preliminary results indicate objective and subjective improvements in gait and balance [12]. However, in some subjects who are relatively well compensated through intensive rehabilitation, there is limited apparent improvement with the BionicVEST—the so-called ceiling-effect. However, even in the well-compensated subjects, it requires a great deal of effort to maintain balance in the absence of vestibular input, resulting in fatigue [13,14,15]. However, this is not easily quantified.

The relationship between cognition and mobility is well known [16,17,18], with dual-task paradigms used to discriminate subjects at higher risk of falls. However, there are no standardized protocols for performing dual-task paradigm testing for balance, and while the results ultimately indicate that impaired cognition is a predictor of falls, the outcome variables are vastly different. Kahya et al. demonstrated that pupillometry can indicate the cognitive effort required for maintenance of posture control in normal participants [19,20]. We hypothesized that pupillometry would be able to capture the increased effort required to maintain posture in subjects with bilateral vestibulopathy in increasingly difficult conditions. Additionally, we hypothesized that the cognitive workload during balance tasks, indexed by pupil size, will decrease with activation of the otolith vestibular implant.

## 2. Materials and Methods

We included 7 subjects who had received a cochleo-vestibular implant (CVI) before March 2023. We excluded subjects who had ophthalmologic conditions or medication use that would preclude pupillometry.

The pupillometry was performed using a modified videonystagmography system (Visual Eyes tm 515/525. Micromedical. Interacoustic, Chatham, IL, USA). Using the VisualEyes goggles, we were able to capture bilateral pupil sizes with eyes opened. We kept the ambient light conditions stable between patients at between 4 and 25 lux (measured with Android mobile app Light Meter—Lux Meter, app version 1.3). For conditions with eyes closed, in lieu of closing the eyes, we placed the blackout cover over the goggles, and the goggles were still able to measure pupil sizes with infra-red. For consistency, we used the videos of the right eye.

Custom-made software was developed using MATLAB (R2008b, Math-Works, Natick, MA, USA) to detect the pupil and to calculate the extent of dilation. This software was validated previously. During the balance tasks, the pupillometry system was synchronized to record the pupil dilation 1 s before the stimuli presentation and up to 2 s after the task completion.

### 2.1. Balance Tasks

While there are many possible tests for balance and posture, we chose to perform the computed dynamic posturography and Modified Clinical Test of Sensory Integration on Balance because we were limited by the length of the cables connecting the Visual Eyes goggles to the computer.

The computed dynamic posturography SMART EquiTest^®^ CDP 8.1 version (NeuroCom^®^ International, Inc., Clackamas, OR, USA) [21] comprises 6 test conditions:

1. Standing with eyes open;
2. Standing with eyes closed;
3. Standing with eyes open with moving visual surroundings;
4. Standing with eyes open with moving platform;
5. Standing with eyes closed with moving platform;
6. Standing with eyes opened with moving visual surroundings and moving platform.

As testing under each condition was performed 3 times, we eliminated the element of surprise and orientation by only taking pupil recordings of the third attempt for each condition.

Modified Clinical Test of Sensory Integration on Balance (mCTSIB):

1. Standing on firm ground with eyes open;
2. Standing on firm ground with eyes closed;
3. Standing on foam with eyes open;
4. Standing on foam with eyes closed.

As test conditions 1 and 2 of the CDP were essentially the same as test conditions 1 and 2 of the mCTSIB, they were omitted during the mCTSIB portion of the testing.

As a control for a non-balance related task requiring cognitive effort, we had the subjects perform the words’ color-naming Stroop task (i.e., naming the color of a word that names a color, the two being incongruent).

### 2.2. Procedure

Subjects were instructed to switch off the vestibular component of their cochleo-vestibular implants for at least 24 h prior to testing. This had been previously tested under closely supervised conditions to be safe for the subjects.

On the day of testing, the entire testing procedure was thoroughly explained, and all questions answered. The subjects wore the goggles while seated, and a 15 s recording of the pupil was taken with the subject resting. The subject then performed the Stroop test with pupil recording. Subjects were then instructed to stand up, obtain their balance, then stand up on the foam with eyes opened, with concurrent pupil recording. After 30 s or whenever the subject fell (while supported), whichever came first, the cover was placed on the goggles while subjects were instructed to keep their eyes open while still balancing on the foam and while pupil recording was continued. After 30 s, or whenever the subject was falling, whichever came first, the task and recordings were stopped.

Subjects then wore the safety harness and performed the CDP. Pupils were recorded for the whole duration of the test, but only the recordings during the 3rd attempts were taken for each test condition.

Subsequently, the vestibular component of the cochleo-vestibular implant was switched on, and subjects went for a 1 h break, and the above test procedure was repeated.

Subjects were able to rest whenever they felt tired.

## 3. Results

There were seven subjects with cochleo-vestibular implants (CVIs). Two were excluded because of visual problems (one subject was blind in one eye, and the other had Cogan syndrome). Another was excluded because he was unable to travel during the short recruitment period. These were the demographics and other characteristics of the four included subjects (Table 1). The CVI was the second side cochlear implant for all subjects (who all had preexisting cochlear implants in the contralateral ear).

For each given task, the lighting was kept constant between subjects. For the Stroop test and mCTSIB, the ambient lux was between 4 and 25. For the computed dynamic posturography, the booth was equipped with its own standard lighting measuring 600–750 lux.

**Figure 1:**
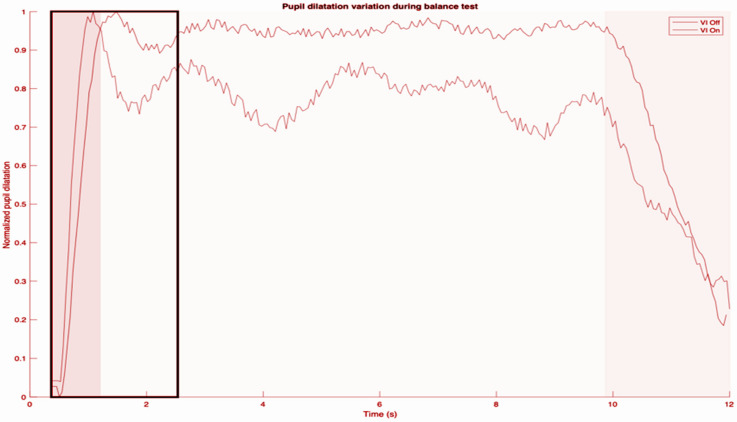
Pupillometry size tracing from a sample subject.

Below is an example of the pupillometry tracing obtained (diameter of pupil) after artifacts such as those due to blinks were processed (Figure 1).

The first dark gray region comprises the basal pupil size and also establishes the minimum diameter for that trace. The highest peak pupil dilation always occurs immediately on commencement of a balance task, which establishes the maximum size. For each subject, we compared all the traces obtained and determined the maximum pupil diameter overall. That maximum pupil diameter was then used to normalize the increase in pupil diameter from basal pupil size. The basal pupil size varied between conditions due to the light conditions (those while doing CDP, and those while doing the mCTSIB). In the light gray region which follows, there were fluctuations in pupil size for the entire duration of the task. However, as demonstrated, they hovered about a mean which was stable for the entire duration of the task. For some, this mean was similar to the first peak pupil dilation, but for others (as in the trace shown), the steady state was at a lower level. Therefore, as a rule, we measured the mean relative pupil diameter (MRPD) one second after the peak pupil dilation. This is supported by basic science studies as well [22]. The final dark gray segment represents the end of the balance task.

**Figure 2:**
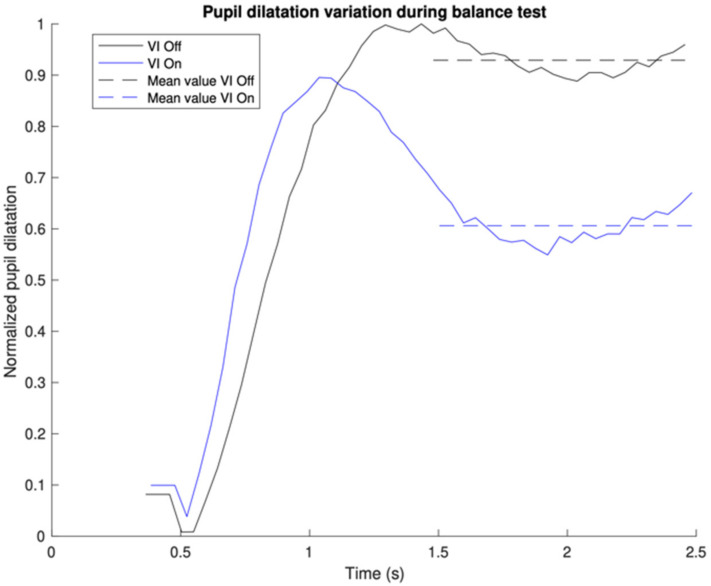
Expanded view of pupillometry size tracing (first shaded area of Figure 1).

Figure 2 focuses only on the first gray region, which is the first one second after the peak pupil dilation, and demonstrates how the MRPD was derived. To compare CVI-off and -on conditions (MRPD difference), we took the MRPD with the CVI-on condition and subtracted it from the MRPD with the CVI-off condition. A positive number indicates that the MRPD with the CVI-off was larger than with the CVI-on. We did not encounter any situation where the MRPD with CVI-on was larger than with CVI-off (Table 2).

During the Stroop test, we obtained consistent and clear recordings of pupil dilations doing color–word discordant tasks, contrasted with minimal pupil dilations when it was concordant, Table 3. This demonstrates that our set up was able to demonstrate the pupil changes related to cognitive effort, serving as a positive control. There were no significant changes (*p* = 0.4187, using the paired *t*-test) in the pupil dilations with implant on and off conditions, which was expected given the Stroop test is not a test of balance. This serves as a negative control.

Due to differences in absolute pupil diameters as well as rates of change due to various factors including age, we used the subject’s own implant-off condition as their own control, and measured the relative differences in pupil size changes.

For the CDP condition 1 (which in our study was collapsed together with mCTSIB condition 1), i.e., standing with eyes open on firm ground, there was little pupil dilation even with implant off, and there was also little change with the implant switched on.

For the CDP condition 2 (which in our study was collapsed together with mCTSIB condition 2), i.e., standing with eyes closed on firm ground, there was an obvious increase in MPRD compared to condition 1 in implant-off condition, and again an obvious reduction in MPRD when the implant was switched on. This relationship was similar for CDP conditions 3 and 4.

For CDP conditions 5 and 6, the amount of pupil dilation with implant off was 82% compared to CDP conditions 3 and 4. When the implant was switched on, there was no significant difference in the pupil sizes.

During mCTSIB condition 3, all subjects were able to complete the task without falling for the full duration of the test when their eyes were open, whether their CVIs were switched on or off. However, pupillometry demonstrated reduced pupil dilations when the CVI was switched on, as seen in Table 2.

During mCTSIB 4, only one subject showed improvement in duration of time balanced on the foam with the CVI switched on (by 8 s), while the rest fell almost immediately with visual input removed, even with the CVI switched on. Pupillometry showed that all had reduced pupil dilation with the CVI switched on, with no trend to indicate which subject had the improved performance (neither the lowest nor the highest reduction in pupillary dilation).

## 4. Discussion

The measurement of pupil sizes during the performance of balance tasks is challenging. In some, there may be a learned response to close their eyes when they are about to fall, almost like a continuation of the blink menace reflex [23]. This can be overcome with clear instructions to keep one’s eyes open, and even repeating the tests; however, it risks fatiguing the subjects.

Indeed, it has been recommended to perform each item of the trial more than 20 times to account for missing data such as contamination or mis-tracking during the assessment of listening effort with pupillometry [24]. However, such numbers are simply impractical for pupillometry during balance tasks due to physical exertion and the resultant fatigue.

Another problem with balance tasks is that losing one’s balance typically evokes strong emotions such as fear. Therefore, the extent of pupil dilation could have contributions both from the effort of trying to maintain one’s balance, but also the fear of falling. It is also important to be able to differentiate a startle response from the shock of a new task, versus pupil dilation due to intense concentration to keep one’s balance. We attempted to minimize the effect of shock by only taking the results from the third attempts on the computed dynamic posturography. For one subject, having learnt that he would be unable to keep his balance based on the first two attempts, he had manifested trepidation with pupil dilations the moment we announced that the third attempt was about to start, even before it had started. Hence, it can be noted that it is difficult to carry out pupillometry when the balance task is overly challenging, due to superimposed fear of falling. However, if we were to omit tasks based on individual capabilities, that would result in missing data points. This would always be a limitation for tests involving a component of psychology.

In addition, it is known that if a task is overly difficult, and the subject simply gives up, there would also be no corresponding pupil changes because the subject has deemed cognitive efforts futile [24,25]. This was clearly demonstrated in our study—during implant-off conditions, there was reduced pupil dilation for CDP conditions 5 and 6 compared to conditions 3 and 4 even though logically it should be the converse. It demonstrates that the subjects had not invested cognitive effort, not that the tasks became easier. We propose that future studies consider the overall capabilities of the study population and eliminate tasks that most subjects would likely fail in, since their performance does not add value.

The clinical effect of the vestibular implant was interesting to observe. When the balance task was easy and the subject had little difficulty even with implant switched off, having the implant switched on minimally reduced the cognitive effort.

In situations where the balance tasks presented higher levels of difficulty (such as in CDP conditions 5 and 6), the observed impact of the vestibular implant seemed limited across all subjects, if compared with previous conditions. However, interpreting the lack of distinction between implant-off and implant-on conditions posed challenges due to the subjects’ apparent lack of motivation, evident particularly in the implant-off scenarios. It is plausible that the implant could have made a difference; however, the subjects might not have been sufficiently motivated to fully engage with the tasks as they could have perceived that the task was too difficult. CDP conditions 5 and 6 are after all challenging, and not very realistic compared to their everyday experiences.

### Study Limitations

The number of patients was limited due to the new approach of this vestibular implant. It is difficult interpreting pupil size changes due to different baseline pupil sizes between light and darkness (eyes-closed scenarios). Therefore, we kept the room lights switched off and the window blinds drawn, achieving conditions as close to darkness as possible whilst still allowing subjects enough light to be able to read (Stroop task) to minimize changes to pupil sizes when they ‘close their eyes’. As the balance task effectively starts immediately when eyes are closed, we mitigate the transition by supporting the subjects for a few seconds after the goggle cover is positioned. The pupil recording only started when the examiner let go of the subjects’ hands.

There are many possible tests for balance and posture; we chose to do the CDP and mCTSIB. This was because we were limited by the length of the cables connecting our goggles to the computer, but also because these tests are standardized, validated, and easily replicated. Moving forward with the advent of wireless videonystagmography goggles, it may be possible to perform pupillometry with a more extended range of tests; however, it would then become a challenge to compare results across different studies.

Another limitation is that our subjects were of varying ages. We know that not only does baseline pupil size decrease with increasing age, but the reactivity decreases as well [26,27,28,29]. While there are attempts at normalizing values based on age in normal subjects [27,30], differences in patient population characteristics means adopting normalized values would be difficult. This contributes also to the lack of consensus on the minimum clinically important difference (MCID) for pupillometry, since these MCID values would vary depending on age as well. It is likely that as pupillometry continues to gain clinical significance, we may reach a consensus on MCID values normalized for various ages.

## 5. Conclusions

The study unveils promising prospects for using pupillometry to quantify the cognitive exertion necessary for individuals with bilateral vestibulopathy to maintain posture. Previous studies had successfully demonstrated a correlation between pupillary changes and the cognitive workload during various balance tasks in normal individuals. This is the first in subjects with bilateral vestibulopathy, and also the first in subjects implanted with a cochleo-vestibular implant, with the resultant small-sample population size.

Activation of the otolith cochleo-vestibular implant notably reduces the cognitive effort required to maintain balance in subjects with bilateral vestibulopathy, particularly in tasks of moderate difficulty. This underscores the implant’s efficacy in aiding posture control. Pupillometry demonstrating a reduction in cognitive effort to maintain posture fills the gap of demonstrating objective improvements in subjects who may have a ‘ceiling effect’ due to the absence of finer objective balance tests.

However, challenges emerged in using pupillometry during these tasks. Issues such as learned responses, emotional influences (like fear of falling), and subject fatigue significantly impact the accuracy of assessing cognitive effort via pupillary changes.

The study’s findings also shed light on the varied effectiveness of the vestibular implant based on task difficulty. Subjects who faced fewer challenges in maintaining balance without the implant exhibited minimal improvement upon its activation. Conversely, in more demanding tasks, the implant’s impact was less apparent, possibly influenced by subject de-motivation with an excessively difficult task.

Future investigations in this domain should focus on standardizing protocols for pupillometry during balance tasks and minimizing confounding factors like emotional responses or excessively challenging task levels beyond the subjects’ capability.

Determining minimum clinically important differences (MCID) normalized for various ages could enhance the clinical significance and interpretation of pupillometry data in similar studies.

In summary, while pupillometry demonstrates promise as a tool to measure cognitive effort in balance tasks among individuals with bilateral vestibulopathy, a further refinement of the methodologies and addressing the challenges are essential for its reliable application in clinical settings and future research endeavors.

## Figures and Tables

**Table 1:** Demographics and characteristics of subjects.

	Subject 1	Subject 2	Subject 3	Subject 4
Age	51	50	65	49
Sex	M	M	M	M
Education Level	Primary school	College and above	Secondary school	College and above
Cause of pathology	Meningitis	Meningitis	Chronic otitis media	Trauma
Duration of deafness before CVI implantation (years)	1 year 5 months	16	15	19
Duration of vestibulopathy before implantation (years)	1 year 5 months	16	5	19
Duration of implant use (months)	55	51	11	20
Laterality of implant	Left	Left	Right	Left
Horizontal canal VHIT gains pre-op (CVI/CI)	0.37/0.12	0.15/0.03	0.21/0.16	0.3/0.09
Horizontal canal VHIT gains 3 months post-op (CVI/CI)	0.28/0.15	0.24/0.27	0.13/0.21	0.05/0.11
CDP composite score pre-op	7	12	30	40
CDP composite 3 months post-op	48	40	45	74
Time up and go pre op (s)	17	10	9	12
Time up and go 3 months post-op (s)	9	9	8	7
DGI pre-op	8	16	17	21
DGI 3 months post-op	19	22	24	24
DHI pre-op	80	28	38	72
DHI 3 months post-op	20	2	36	58

Abbreviations: M—male; CVI—cochleo-vestibular implant. VHIT—video head impulse test; CI—cochlear implant; CDP—computed dynamic posturography; DGI—dynamic gait index; DHI—dizziness handicap index.

**Table 2:** Improvement in duration subjects can maintain balance on foam with lights on and off (mCTSIB 3 and 4) with the activation of the CVI.

Gain in Time Spent on Foam	Condition	Subject 1	Subject 2	Subject 3	Subject 4
(CVI On)—(CVI Off)	Light On	0	0	0	0 *
(seconds)	Light Off ^+^	0	1	8	0

* In lights on condition, zero seconds in all 4 subjects, indicates no difference in time maintaining balance before falling between CVI-on and -off states. This is because all were able to maintain their balance for the full 30 s. ^+^ Lights off condition, i.e., goggle cover put on. We measured the time before the subject lost balance with the CVI off and subtract it from the time with CVI on. Positive results show that subjects maintained their balance for longer with CVI on.

**Table 3:** Mean relative pupillary dilation (MRPD) for all subjects.

Condition	Subject 1	Subject 2	Subject 3	Subject 4
Concordant vs. Stroop (CVI Off)	39.2%	18.5%	19.5%	30%
Concordant vs. Stroop (CVI On)	18.6%	18.5%	20.6%	29.9%
mCTSIB Cond 3	25.5%	5.7%	17.8%	25.6%
mCTSIB Cond 4	36.5%	19.2%	22.8%	15.4%
CDP Cond 1	7%	16.2%	4.9%	14.7%
CDP Cond 2	27.1%	31.9%	26.9%	10.5%
CDP Cond 3	28.6%	20.9%	23.4%	23.6%
CDP Cond 4	30.7%	22.6%	27.9%	9.8%
CDP Cond 5	11%	16.6%	9.7%	10.4%
CDP Cond 6	12.4%	9.4%	13.9%	7.9%

## Data Availability

The data presented in this study are available on request from the corresponding author. The data are not publicly available due to privacy.
